# The *Salmonella enterica* serovar Typhimurium virulence factor STM3169 is a hexuronic acid binding protein component of a TRAP transporter

**DOI:** 10.1099/mic.0.000967

**Published:** 2020-09-07

**Authors:** Reyme Herman, Cavan Bennett-Ness, Abbas Maqbool, Amna Afzal, Andrew Leech, Gavin H. Thomas

**Affiliations:** ^1^​ Department of Biology, University of York, Wentworth Way, York YO10 5DD, UK; ^‡^​ Present address: Institute of Genetics and Molecular Medicine, University of Edinburgh WGH, Crewe Road South, Edinburgh EH4 2XU, UK; ^§^​ Present address: The Sainsbury Laboratory, Norwich NR4 7UH, UK

**Keywords:** D-glucuronic acid, hexuronic acid, *Salmonella enterica*serovar Typhimurium, STM3169, TRAP transporter

## Abstract

The intracellular pathogen *S*. Typhimurium is a leading cause of foodborne illness across the world and is known to rely on a range of virulence factors to colonize the human host and cause disease. The gene coding for one such factor, *stm3169*, was determined to be upregulated upon macrophage entry and its disruption reduces survival in the macrophage. In this study we characterize the STM3169 protein, which forms the substrate binding protein (SBP) of an uncharacterized tripartite ATP-independent periplasmic (TRAP) transporter. Genome context analysis of the genes encoding this system in related bacteria suggests a function in sugar acid transport. We demonstrate that purified STM3169 binds d-glucuronic acid with high affinity and specificity. *S*. Typhimurium LT2 can use this sugar acid as a sole carbon source and the genes for a probable catabolic pathway are present in the genome. As this gene was previously implicated in macrophage survival, it suggests a role for d-glucuronate as an important carbon source for *S*. Typhimurium in this environment.

## Introduction

Non-typhoidal *Salmonella* is a leading cause of foodborne illness worldwide [1]. In particular, *Salmonella enterica* serovar Typhimurium (*S*. Typhimurium) is a common cause of gastroenteritis and can lead to inflammatory diarrhoea and bacteraemia [2, 3]. This intracellular pathogen is able to survive and replicate within both phagocytic and non-phagocytic cells. The intracellular environment of a macrophage phagosome is harsh, containing acid, bactericidal compounds and reactive oxygen species [4, 5]. However, this also provides a niche for adaptive enteropathogenic bacteria such as *S*. Typhimurium to colonize and replicate within, isolated from patrolling immune cells [6]. These bacteria rely on a global change in gene expression to adapt to the intracellular environment, employing a range of virulence factors to mediate host cell entry, survival and growth.

Haneda *et al.* investigated the shift in protein levels in *S.* Typhimurium strain SH100 upon macrophage entry and identified over 50 proteins whose increased accumulation was dependent on the stringent response-related signalling molecule ppGpp [7]. The gene encoding the most differentially regulated protein, *stm3169*, was then disrupted on the *S.* Typhimurium chromosome, and the resulting strain had a significant growth defect inside macrophage-like RAW264.7 cells [7]; as such, STM3169 was suggested to be important for macrophage survival and virulence.

In this study we have characterized the function of this protein, which encodes a substrate binding protein of an uncharacterized tripartite ATP-independent periplasmic (TRAP) transporter. We provide a novel perspective into important cellular functions needed for macrophage survival.

## Methods

### Cloning, expression and purification of STM3169

The gene *stm3169* was amplified from *S*. Typhimurium LT2 (ATCC 700720) and cloned into pETYSBLIC3C [8] by ligation-independent cloning. This plasmid was transformed into the expression host *Escherichia coli* BL21 (DE3). Protein expression was carried out in 1 litre of Luria Bertani (LB) medium supplemented with kanamycin at 50 μg ml^−1^, with 0.4 mM IPTG added once the optical density (OD_600_) reached 0.6. Cells were incubated at 37 °C at 120 r.p.m. overnight, then harvested by centrifugation at 7800 ***g*** for 25 min. The cell pellet was resuspended in wash buffer (50 mM KPi pH 7.8, 200 mM NaCl, 20 % glycerol, 40 mM imidazole) containing 0.4 mM AEBSF protease inhibitor (Sigma Aldrich). The sample was sonicated at 70 W for 3 min, followed by centrifugation at 27 000 ***g*** at 4 °C for 30 min. Ni^2+^ affinity chromatography was used to purify the His_6_-tagged protein. The cell lysate was loaded onto a HisTrap column (GE Healthcare) and washed with the aforementioned wash buffer, then eluted with the elution buffer (50 mM KPi pH 7.8, 200 mM NaCl, 20 % glycerol, 500 mM imidazole). The protein of interest was identified by SDS-PAGE. The purified protein was buffer exchanged into 50 mM KPi pH 7.8 and 200 mM NaCl using the HiTrap Desalting column (GE Healthcare). Protein concentration was measured by determining the absorbance at 280 nm and using the Beer–Lambert law.

### Protein renaturation

A fraction of the desalted protein was then loaded onto a 5 ml HisTrap column (GE Healthcare). The column was then washed with 30 column volumes (CV) of denaturation buffer (50 mM KPi pH 7.8, 200 mM NaCl, 20 % glycerol, 40 mM imidazole, 2 M guanidine hydrochloride) to unfold the bound protein. A gradient wash across 12 CV was performed from the denaturation buffer to the previously mentioned guanidine hydrochloride-free wash buffer to refold the protein. A further 8 CV wash using the wash buffer was performed before elution using the elution buffer as before. Circular dichroism was used to check correct refolding of the renatured protein. CD spectra were determined using a J810 spectropolarimeter. Protein samples were dialysed into 20 mM KPi pH 7.8 buffer and diluted to a concentration of 0.2 mg ml^−1^. The spectra were determined in a 1 mm pathlength quartz cuvette between 260 and 180 nm at 100 nm min^–1^ with 1 nm pitch. Prior to any downstream experiments, the protein was desalted into 50 mM KPi pH 7.8 and 200 mM NaCl. The concentration of the refolded protein was measured by determining the absorbance at 280 nm and using the Beer–Lambert law and predicted extinction coefficient from ProtParam (Expasy).

### Thermal shift assay

The Protein Thermal Shift Dye kit (Applied Biosystems) was used to carry out the thermal shift assay. A final concentration of 5 µM protein in 50 mM KPi, 20 mM NaCl and each ligand at final concentrations of 1 nM, 1 µM and 1 mM was added to the thermal shift dye in triplicate. The experiments were performed using a StepOne Real-Time PCR system (Applied Biosystems) with a temperature ramp of 1.5 °C every 2 min from 25 to 99 °C. The melting temperatures (*T*_m_) were calculated by determining the inflection point of the plot of the normalized fluorescence measurements against temperature.

### Intrinsic fluorescence spectroscopy

All intrinsic fluorescence measurements were performed using a FluoroMax 4 fluorescence spectrometer (Horiba Jobin-Yvon). STM3169 in 50 mM KPi pH 7.8 plus 200 mM NaCl buffer was used at a concentration of 0.5 µM. The protein was excited at 297 nm with slit widths of 3.5 nm and emission was monitored at 330 nm. To test for potential ligands, various sugar acids were sequentially added at concentrations of 100 µM.

To quantitatively assess binding of specific ligands, protein was titrated with increasing concentrations of ligand in order to elucidate the *K*_D_of ligand binding. Cumulative fluorescence was then plotted in Prism 5 (GraphPad) and the *K*_D_ was calculated from the hyperbolic fit of the binding curve.

### Microscale thermophoresis

The fluorescent dye NT-647-NHS was used at 30 µM to label 15 µM of STM3169. Any unreacted dye was removed using a HiTrap Desalting column (GE Healthcare). Protein and dye concentrations were measured by absorbance at 280 and 650 nm, respectively. The concentration of ligand was varied from 200 µM to 5 nM in a 16-step dilution, then mixed with labelled protein in capillaries for data acquisition using the Monolith NT.115 (NanoTemper). Outliers were detected using the NanoTemper analysis software.

### Mass spectrometry by FT-ICR-MS

The natively folded STM3169 containing an N-terminal tag (MGSSHHHHHHSSGLEVLFEGPA) attached to the mature peptide was used. A sample of this protein was dialysed into 35 mM ammonium acetate pH 5 and diluted to a final protein concentration of 100 µM. Fourier-transform ion cyclotron resonance MS (FT-ICR-MS) was performed using a solariX FT-MS device (Bruker Daltonics) with a 9.4-T superconducting magnet. Analysis was performed in denaturing conditions where the protein was diluted to 1 µM in 50 % (v/v) acetonitrile containing 0.1 % (v/v) formic acid before introduction via a TriVersa NanoMate (Advion BioSciences) source in positive-ion mode. The applied voltage was adjusted to 1.4–1.7 kV to achieve a stable ion current. A 120 °C nitrogen dry gas was supplied at 1.3 l min^–1^ to aid desolvation. Instrument control and data acquisition used Compass 1.4 (Bruker Daltonics). Spectra were generated by the accumulation of 20 scans with 0.1 s ion cooling time and 0.4 s scan time with 8 million data points recorded. To improve resolution and reduce space-charge effects, the 42+ protein charge states were isolated in the hexapole before scanning in the FT-ICR cell. Spectra from isolated charge states were deconvoluted to monoisotopic protein mass using version 2.0 of the SNAP averaging algorithm (C 4.9384, N 1.3577, O 1.4773, S 0.0417 and H 7.7583 %), in DataAnalysis version 4.0 (Bruker Daltonics).

### Homology modelling of STM3169

The predicted structure of the mature STM3169 was prepared by automated homology modelling on SWISS-MODEL (swissmodel.expasy.org) [9–12]. The structure of Bamb_6123 from *Burkholderia ambifaria* AMMD (PDB ID: 4N15) was used as a template for homology modelling of the mature peptide of STM3169. The QMEAN Z-score was predicted to be −0.69, which suggests that the model of STM3169 is consistent with other experimentally determined structures of a similar size. The derived GMQE (global model quality estimation) for the model was determined to be 0.74, indicating high reliability.

### *S*. Typhimurium LT2 growth experiments

A stationary phase culture of *S.* Typhimurium LT2 (ATCC 700720) grown in Luria Bertani (LB) media was centrifuged and washed with M9 salts [13] to remove any residual LB. These were inoculated into 96-well microplates with an initial OD_600_ of 0.01 in M9 media (48 mM Na_2_HPO_4_, 22 mM KH_2_PO_4_, 19 mM NH_4_Cl, 9 mM NaCl, 2 mM MgSO_4_) with 5 mM carbon source (d-glucose, d-galactose, sodium d-glucuronate, sodium d-galactonate, l-arabinose; Sigma Aldrich). Triplicate cultures were grown in the microplates at 37 °C, and shaken in an Epoch2 microplate spectrophotometer (BioTek). OD_600_ measurements were taken every hour for 15 h.

## Results and discussion

**Fig. 1.**
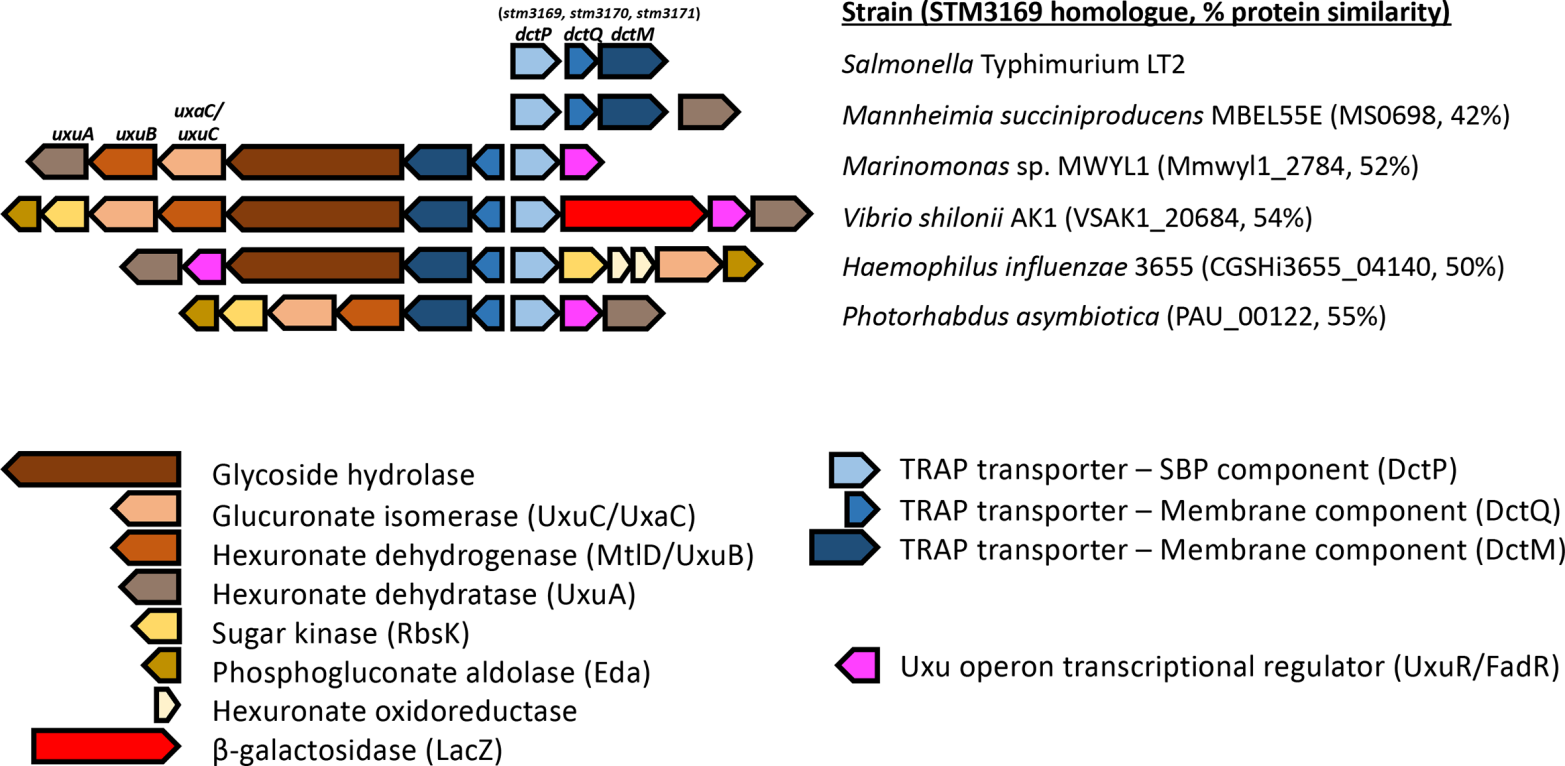
Homologues of STM3169 may be encoded from operons containing hexuronate metabolic genes. In *S*. Typhimurium LT2, *stm3169* is found on an operon with *stm3170* and *stm3171*, the two putative membrane components of a TRAP transporter. This operon is not surrounded by other genes that might be involved in uptake or sugar metabolism. Some genes neighbouring *stm3169* homologues were predicted to code for the hexuronate catabolism enzymes UxuAB and a glucuronate isomerase, UxuC/UxaC. Genes are represented by coloured bars, identifying its encoded protein’s putative function. Homologues were identified in other strains using MicrobesOnline.

The *stm3169* gene from *S.* Typhimurium LT2 is encoded within a typical operon for a TRAP transporter, in that it also includes genes encoding the two required membrane subunits (*stm3170* and *stm3171*) [14] and so is probably part of a functioning transporter (Fig. 1). STM3169 is the substrate binding protein (SBP) component that initially binds the substrate in the periplasm; this component is usually expressed at high levels relative to the membrane domains, consistent with the high abundance of the STM3169 protein in the Haneda *et al.* study [7, 14].

TRAP transporters form a large family of diverse SBP-dependent secondary transporters in bacteria, the functions of which are, largely, to move organic acids into bacterial cells [15–17]. A hallmark sign of this substrate preference is a conserved arginine residue in the SBP subunit that coordinates the carboxylate group of the acid ligand [18, 19]. This residue is conserved in STM3169 (Arg170), suggesting a typical organic acid-type ligand. Beyond predicting that the ligand contains a carboxylate group, the binding sites of different TRAP transporter SBPs are highly variable, allowing them to accommodate ligands of many different sizes and shapes, often using complex water networks [17, 20].

In *S.* Typhimurium LT2, the *stm3169–stm3171* TRAP transporter gene cluster is not found in a region containing any other associated genes that give any clue to its substrate and hence cellular function (Fig. S1a, available in the online version of this article). However, genome analysis of *stm3169* orthologues from related gamma-proteobacteria revealed a range of different cluster architectures that strongly suggest a role in sugar acid import and catabolism for these strains (Fig. 1). A common feature of these clusters is the presence of genes coding for proteins predicted to be involved in hexuronate catabolism. These proteins, UxuA, UxuB and UxuC, have been characterized in *Erwinia chrysanthemi* where they are responsible for the utilization of d-glucuronate following uptake by the hexuronate MFS transporter ExuT [21]. The presence of homologues of UxuABC (STM3135–STM3137) with ExuT (STM3134) in *S*. Typhimurium (Fig. S1b) suggests its ability to catabolize this sugar. Additionally, this pattern of co-occurrence is found in other genomes represented in MicrobesOnline [22], suggesting that this TRAP transporter might be involved in uptake of hexuronic acids.

To test this hypothesis, we expressed and purified recombinant STM3169 from *E. coli* using nickel-affinity chromatography (Fig S2a). A portion of the purified protein was denatured using guanidine hydrochloride and refolded, removing any endogenous ligand potentially carried over during the purification, a common phenomenon with these SBPs [23, 24]. Analysis of the protein by circular dichroism suggested minimal differences in the folding when comparing the native and refolded protein, suggesting successful protein refolding (Fig. S2b). The protein was analysed by MS to have a mass of 35 741.96 Da, which matches the predicted monoisotopic mass of the protein (Fig. S2c). An additional signal at mass 35 840.4 was observed, which, being 98 Da greater than the predicted signal, is probably a single phosphate adduct on the protein, an artefact we have observed with other SBPs [25].

**Fig. 2.**
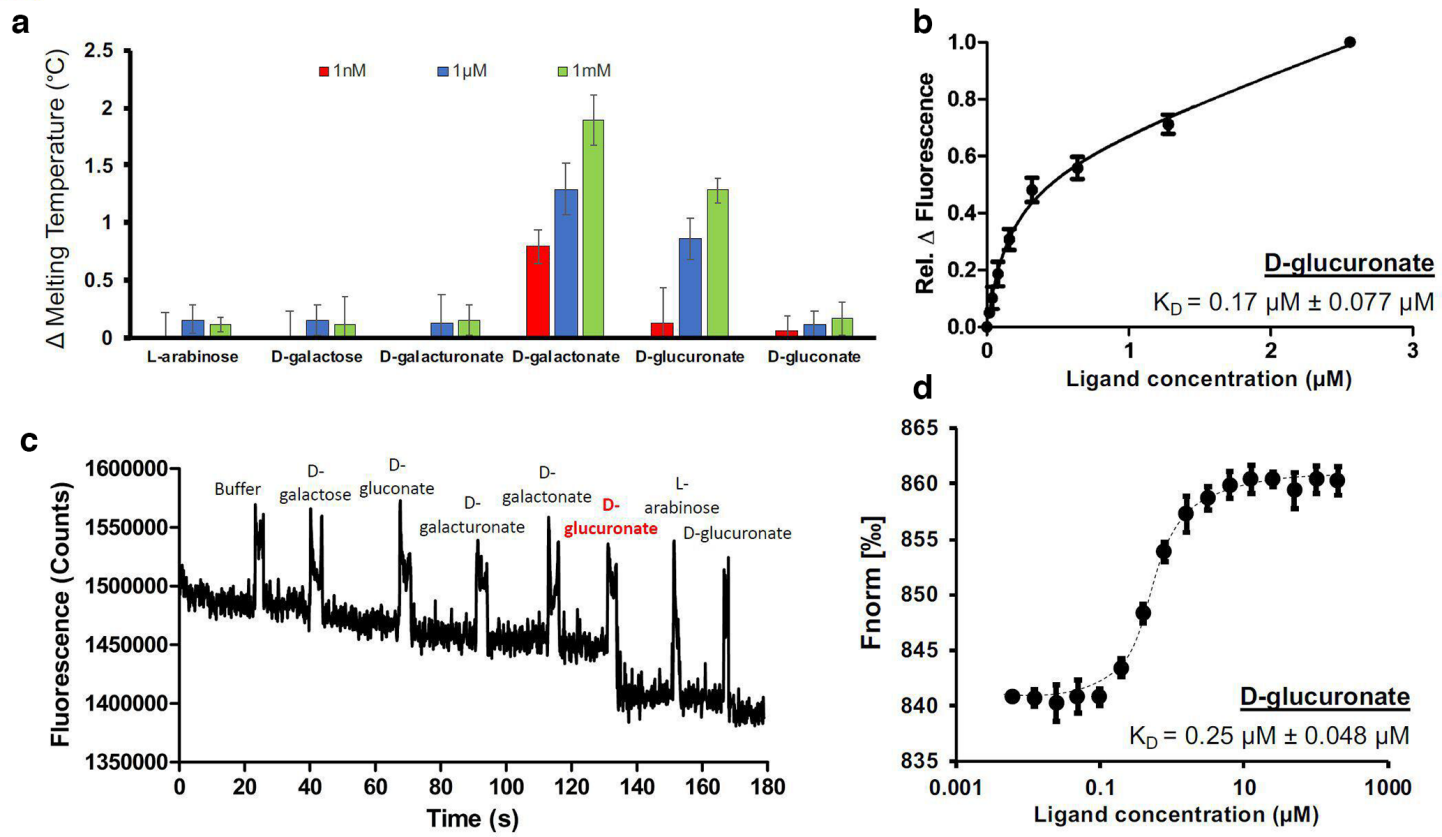
STM3169 binds to d-glucuronate with high affinity. The refolded protein was tested against five hexoses (d-galactose, d-galacturonate, d-galactonate, d-glucuronate, d-gluconate) and a pentose sugar, l-arabinose, as a negative control by (a) thermal shift assays and (b) measuring changes in intrinsic fluorescence. The binding affinity (*K*_D_) of d-glucuronate to STM3169 was then determined by titration using (c) relative changes in intrinsic fluorescence and further supported by (d) microscale thermophoresis. Data from (a), (c) and (d) are from three independent repeats and error bars indicate ±sd.

To screen potential substrates for this TRAP transporter we used an established fluorophore-based protein thermal shift assay [17] using a range of monosaccharides and their related acid forms. Analysis of the natively purified protein showed no significant changes in protein thermal stability in the presence of any of the compounds tested: d-glucuronate, d-galacturonate, d-gluconate, d-galactonate, d-galactose and the negative control, l-arabinose (data not shown). However, for the refolded and presumably ligand-free protein, increases in protein thermal stability were observed in the presence of two sugar derivatives, d-galactonate and d-glucuronate (Fig. 2a). These data suggest that the purified protein from *E. coli* came with pre-bound ligand and also suggested ligands for continuation of *in vitro* binding experiments.

To further assess binding we used fluorescence spectroscopy, measuring the change in intrinsic protein fluorescence upon ligand binding. Using protein excitation at 295 nm, the same set of sugar acids were added sequentially to refolded STM3169, detecting fluorescence in time acquisition mode. Whilst addition of d-galactonate had little effect on fluorescence, d-glucuronate caused an ~10 % quench in fluorescence (Fig. 2b). This discrepancy may be attributed to the nature of protein thermal shift experiments as non-specific interactions could give rise to a shift in thermal stability.

We proceeded to determine the binding affinity of STM3169 for d-glucuronate by titration and measuring the resulting changes in intrinsic fluorescence (Fig. 2c). The experimental data suggested d-glucuronate was binding to STM3169 with a binding constant (*K*_D_) of 0.17±0.077 µM. As a complementary biophysical methodology, we examined this interaction using microscale thermophoresis (MST), measuring a ligand-dependent change in thermophoresis of dyed protein molecules to indicate conformational changes that accompany ligand binding. This technique also suggested d-glucuronate was binding STM3169, and through repeated 16-step titrations a *K*_D_ of 0.25±0.048 µM was estimated (Fig. 2d). No clear signal suggesting a conformational change upon d-galactonate binding was observed (data not shown). Together, these data are consistent with d-glucuronate being a ligand for STM3169, with affinities in the range determined for other high-affinity TRAP transporters such as the SBP of the sialic acid transporter of *Haemophilus influenzae *, SiaP, with a reported *K*_D_ of ~0.1 µM [26].

**Fig. 3.**
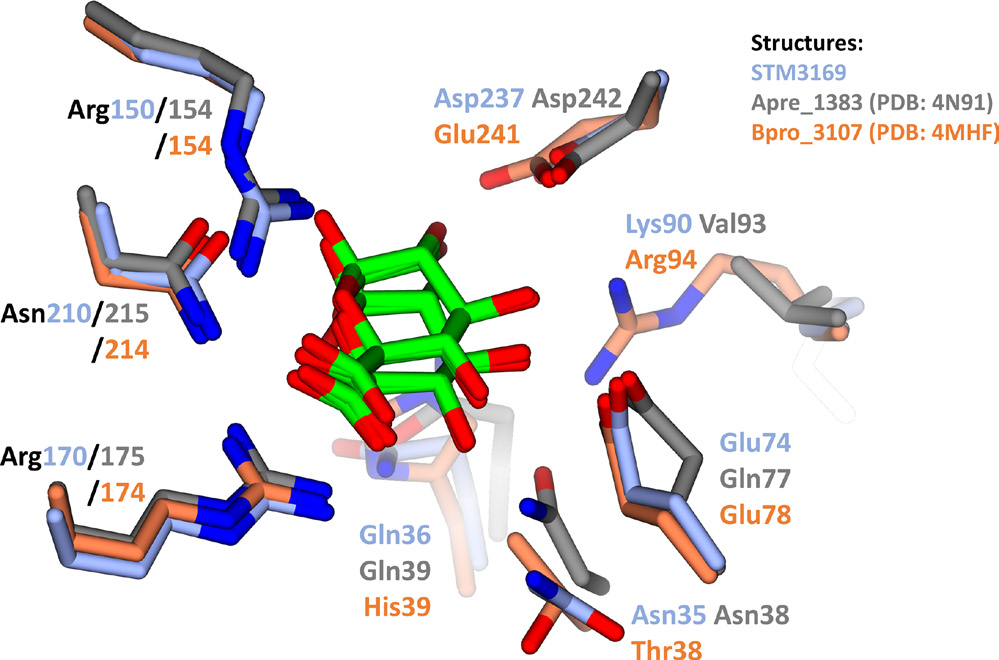
The putative binding site of STM3169 resembles that of characterized d-glucuronate binding proteins. Residues which were experimentally shown to contribute to ligand binding and their various homologous residues were identified. The carbon atoms and residue names of the various homologous residues were coloured by individual proteins. The d-glucuronate binding protein Apre_1383 from *Anaerococcus prevotii* PC1 (4N91, grey), the d-glucuronate and d-galacturonate binding protein Bpro_3107 from *Polaromonas* sp. JS666 (4MHF, orange) are shown. STM3169 from *S*. Typhimurium LT2 (light blue) was modelled using the crystal structure of Bamb_6123 (4N15) on SWISS-MODEL (swissmodel.expasy.org). Carbon atoms of the ligands, the alpha and beta anomers of d-glucuronate, are represented in light green. Nitrogen and oxygen atoms in the various structures are in blue and red, respectively. Residue names denoted in black are conserved between all structures analysed.

A recent study by Vetting *et al.* [17] screened 158 SBPs against a large number of potential ligands to classify these proteins into isofunctional clusters. A number of these SBPs were then characterized structurally, some of which were successfully co-crystallized with d-glucuronate. We sought to compare these crystal structures against a model of STM3169 prepared on SWISS-MODEL (swissmodel.expasy.org) [9–12] to supplement our *in vitro* findings. These proteins were superposed using the secondary-structure matching (SSM) algorithm [27] on CCP4MG [28] and the measured root mean square distances of the alignments were found to be between 1 Å and 1.56 Å, suggesting highly similar overall structures. Analysis of the aligned binding sites (Fig. 3) initially revealed two conserved arginines (Arg150, Arg170 in STM3169) and an asparagine (Asn210 in STM3169), consistent with known d-glucuronate binding proteins [18]. As predicted, Arg170 in the model of STM3169 aligns with the residues responsible for coordinating the carboxyl group of the ligand in the d-glucuronate bound structures of Apre_1383 and Bpro_3107. The two other conserved residues, Arg150 and Asn210, have been shown to further coordinate the ligand by forming multiple hydrogen interactions. STM3169 residues Asn35, Gln36, Glu74 and Asp237 are fully conserved in the d-glucuronate-specific binding protein Apre_1383 and its corresponding residues form more hydrogen bonds with d-glucuronate. Bpro_3107, a d-glucuronate and d-galacturonate binding protein, possesses alternative residues also capable of coordinating the ligand. This intricate network of multiple predicted hydrogen bonds is likely to give rise to the high binding affinity for d-glucuronate shown though our *in vitro* assays.

**Fig. 4.**
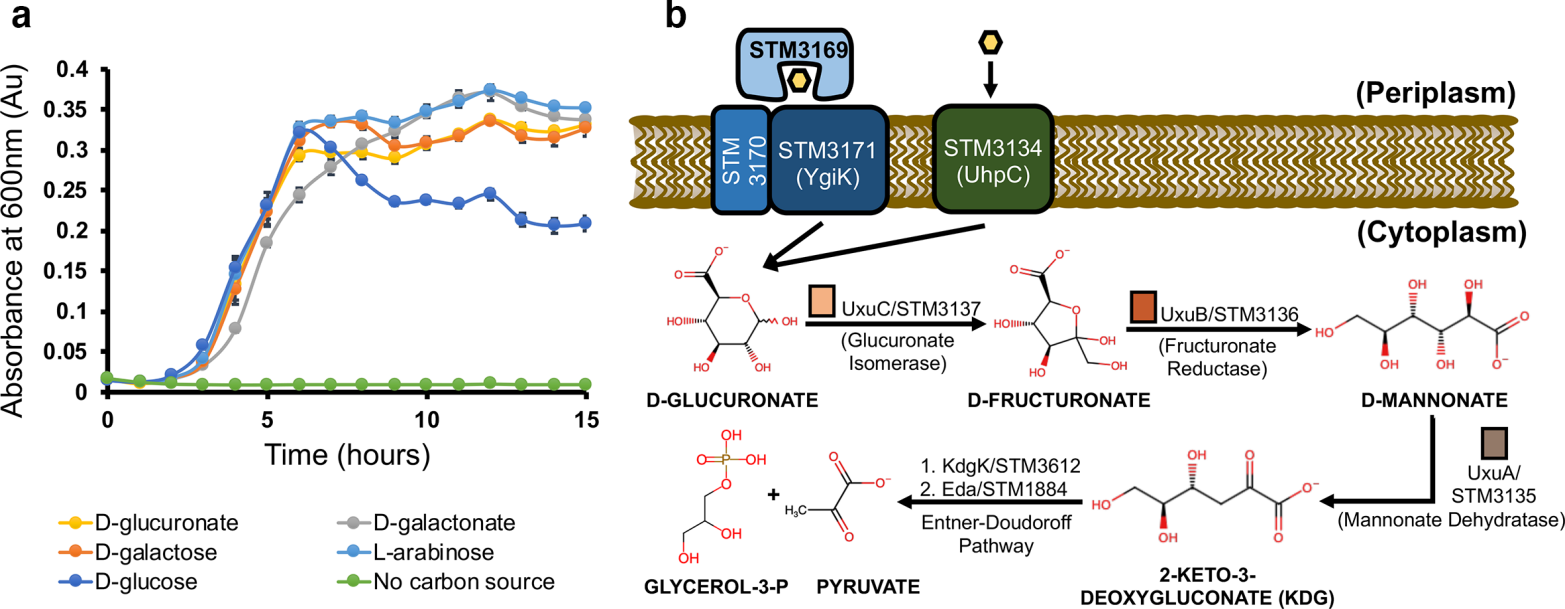
d-Glucuronate serves as a carbon source for *S*. Typhimurium. (a) *S*. Typhimurium LT2 was grown in minimal media using d-glucose, d-glucuronate, d-galactose, d-galactonate and l-arabinose as the sole carbon source. Data points are the average of three independent replicates and error bars indicate ±sd. (b) Proposed model for the uptake of d-glucuronate. d-Glucuronate can potentially be transported by the STM3169–STM3171 TRAP transporter system or the putative hexuronate transporter STM3134. Once inside the cell, UxuABC converts d-glucuronate to 2-keto-3-deoxygluconate (KDG)_ and, via the Entner–Doudoroff pathway, the latter is broken down into glycerol-3-phosphate and pyruvate for use. Coloured bars correspond to the various representations on Fig. 1.

Through *in vitro* studies and homology modelling, we have characterized a high-affinity substrate binding component of the TRAP transporter with apparent specificity for d-glucuronate. As *S.* Typhimurium also has homologous genes coding for proteins involved in d-glucuronate catabolism, it should be able to import d-glucuronate and utilize it as a carbon source. We confirmed this, an observation first observed in 1969 from the Ames group [29] (Fig. 4a); furthermore, we demonstrated the utilization of a selection of other sugars used in this study. From these data we propose a model (Fig. 4b) whereby the STM3169 protein forms part of a functioning TRAP transporter that functions alongside the putative UhpC/ExuT hexuronate transporter (STM3134) that is divergently transcribed from the *uxuABC* genes (*stm3135–stm3137*). After transport, the bacterium would use the UxuABC proteins and the enzymes of the Entner–Doudoroff pathway to convert d-glucuronate into central metabolites. The high-affinity nature of the TRAP transporter, compared to the lower affinity major facilitator superfamily protein ExuT, might suggest that d-glucuronate is present in the macrophage as a carbon source but at low micromolar concentrations, which would explain the significant increase in protein levels in the macrophage to ensure its efficient uptake [7].

This ability to metabolize sugar acids is key for the colonization of intracellular niches, where other substrates for glycolysis may not be present [16]. The landmark study of Eriksson *et al.* [30], which provided the first comparison of *S*. Typhimurium transcriptional changes upon moving to the intravacuolar environment of a macrophage, revealed that the organism appeared to be using the related sugar acid d-gluconate and the Entner–Douderoff pathway during growth [30]. In fact, the UhpT/ExuT-like transporter STM3134 was identified as being specifically upregulated as well as *dgoK* and *dgoA*, which encode enzymes that interconvert the UxuABC reaction products into pyruvate and glyceraldehyde-3-phosphate, suggesting a broader role for sugar acid molecules as metabolites. While STM3134 may be responsible for the transport of multiple hexuronate carbon sources, we have demonstrated that STM3169–STM3171 is a selective high-affinity transport system for d-glucuronate.

Despite the lack of evidence to suggest that the STM3169–STM3171 TRAP transporter is able to transport d-galactonate, our defined growth media assay (Fig. 4a) has hinted towards another transporter capable of efficient d-galactonate uptake. This and other sugars found in the macrophage could be taken up by other high-affinity transporters, including further members of the TRAP family. There is previous evidence suggesting the two other members of the TRAP family in *S*. Typhimurium LT2, STM3671–STM3673 and STM4052–STM4054, have transport functions which could include carbon sources in the macrophage. The SBP of STM3671–STM3673 is 88 % identical to the previously characterized *E. coli* 2,3-diketo-l-gulonate binding protein, YiaO [31–33], whilst STM4052–STM4054 is thought to be coregulated with proteins of the rhamnose utilization pathway by the activator RhaS (STM4048) through promoter analysis by RegPrecise [34].

Finally, we have shown evidence of function for the long-identified virulence protein STM3169 and suggested a role for its associated transport system in cell metabolism. Our discovery adds to our understanding of how pathogenic bacteria such as *S.* Typhimurium are able to survive within hostile host intracellular environments. Given its key role in virulence as observed by Haneda *et al.* [7], this knowledge could potentially aid in the design of competitive inhibitors.

## Supplementary Data

Supplementary material 1

## References

[1] WHO (2018). Salmonella (Non-Typhoidal). World Health Organization.

[2] Majowicz SE, Musto J, Scallan E, Angulo FJ, Kirk M (2010). The global burden of nontyphoidal *Salmonella gastroenteritis*. Clin Infect Dis.

[3] Laupland KB, Schønheyder HC, Kennedy KJ, Lyytikäinen O, Valiquette L (2010). *Salmonella enterica* bacteraemia: a multi-national population-based cohort study. BMC Infect Dis.

[4] Sierra H, Cordova M, Chen C-SJ, Rajadhyaksha M (2015). Confocal imaging-guided laser ablation of basal cell carcinomas: an *ex vivo* study. J Invest Dermatol.

[5] Torraca V, Masud S, Spaink HP, Meijer AH (2014). Macrophage-pathogen interactions in infectious diseases: new therapeutic insights from the zebrafish host model. Dis Model Mech.

[6] Monack DM, Bouley DM, Falkow S (2004). *Salmonella* Typhimurium persists within macrophages in the mesenteric lymph nodes of chronically infected Nramp1+/+ mice and can be reactivated by IFNgamma neutralization. J Exp Med.

[7] Haneda T, Sugimoto M, Yoshida-Ohta Y, Kodera Y, Oh-Ishi M (2010). Comparative proteomic analysis of *Salmonella enterica* serovar Typhimurium ppGpp-deficient mutant to identify a novel virulence protein required for intracellular survival in macrophages. BMC Microbiol.

[8] Fogg MJ, Wilkinson AJ (2008). Higher-throughput approaches to crystallization and crystal structure determination. Biochem Soc Trans.

[9] Waterhouse A, Bertoni M, Bienert S, Studer G, Tauriello G (2018). SWISS-MODEL: homology modelling of protein structures and complexes. Nucleic Acids Res.

[10] Bienert S, Waterhouse A, de Beer TAP, Tauriello G, Studer G (2017). The SWISS-MODEL Repository-new features and functionality. Nucleic Acids Res.

[11] Guex N, Peitsch MC, Schwede T (2009). Automated comparative protein structure modeling with SWISS-MODEL and Swiss-PdbViewer: a historical perspective. Electrophoresis.

[12] Benkert P, Biasini M, Schwede T (2011). Toward the estimation of the absolute quality of individual protein structure models. Bioinformatics.

[13] Neidhardt FC, Bloch PL, Smith DF (1974). Culture medium for enterobacteria. J Bacteriol.

[14] Kelly DJ, Thomas GH (2001). The tripartite ATP-independent periplasmic (TRAP) transporters of bacteria and archaea. FEMS Microbiol Rev.

[15] Mulligan C, Fischer M, Thomas GH (2011). Tripartite ATP-independent periplasmic (TRAP) transporters in bacteria and archaea. FEMS Microbiol Rev.

[16] Rosa LT, Bianconi ME, Thomas GH, Kelly DJ (2018). Tripartite ATP-independent periplasmic (TRAP) transporters and tripartite tricarboxylate transporters (TTT): from uptake to pathogenicity. Front Cell Infect Microbiol.

[17] Vetting MW, Al-Obaidi N, Zhao S, San Francisco B, Kim J (2015). Experimental strategies for functional annotation and metabolism discovery: targeted screening of solute binding proteins and unbiased panning of metabolomes. Biochemistry.

[18] Fischer M, Hopkins AP, Severi E, Hawkhead J, Bawdon D (2015). Tripartite ATP-independent periplasmic (TRAP) transporters use an arginine-mediated selectivity filter for high affinity substrate binding. J Biol Chem.

[19] Fischer M, Zhang QY, Hubbard RE, Thomas GH (2010). Caught in a TRAP: substrate-binding proteins in secondary transport. Trends Microbiol.

[20] Darby JF, Hopkins AP, Shimizu S, Roberts SM, Brannigan JA (2019). Water networks can determine the affinity of ligand binding to proteins. J Am Chem Soc.

[21] Hugouvieux-Cotte-Pattat N, Robert-Baudouy J (1987). Hexuronate catabolism in *Erwinia chrysanthemi*. J Bacteriol.

[22] Dehal PS, Joachimiak MP, Price MN, Bates JT, Baumohl JK (2010). MicrobesOnline: an integrated portal for comparative and functional genomics. Nucleic Acids Res.

[23] Horler RSP, Müller A, Williamson DC, Potts JR, Wilson KS (2009). Furanose-specific sugar transport: characterization of a bacterial galactofuranose-binding protein. J Biol Chem [Internet].

[24] Maqbool A, Levdikov VM, Blagova EV, Hervé M, Horler RSP (2011). Compensating stereochemical changes allow murein tripeptide to be accommodated in a conventional peptide-binding protein. J Biol Chem.

[25] Horler RSP, Müller A, Williamson DC, Potts JR, Wilson KS (2009). Furanose-specific sugar transport: characterization of a bacterial galactofuranose-binding protein. J Biol Chem.

[26] Severi E, Randle G, Kivlin P, Whitfield K, Young R (2005). Sialic acid transport in *Haemophilus influenzae* is essential for lipopolysaccharide sialylation and serum resistance and is dependent on a novel tripartite ATP-independent periplasmic transporter. Mol Microbiol.

[27] Krissinel E, Henrick K (2004). Secondary-structure matching (SSM), a new tool for fast protein structure alignment in three dimensions. Acta Crystallogr D Biol Crystallogr.

[28] McNicholas S, Potterton E, Wilson KS, Noble MEM (2011). Presenting your structures: the CCP4mg molecular-graphics software. Acta Crystallogr D Biol Crystallogr.

[29] Gutnick D, Calvo JM, Klopotowski T, Ames BN (1969). Compounds which serve as the sole source of carbon or nitrogen for Salmonella typhimurium LT-2. J Bacteriol.

[30] Eriksson S, Lucchini S, Thompson A, Rhen M, Hinton JCD (2003). Unravelling the biology of macrophage infection by gene expression profiling of intracellular Salmonella enterica. Mol Microbiol.

[31] Thomas GH, Southworth T, León-Kempis MR, Leech A, Kelly DJ (2006). Novel ligands for the extracellular solute receptors of two bacterial TRAP transporters. Microbiology.

[32] Plantinga TH, van der Does C, Tomkiewicz D, van Keulen G, Konings WN (2005). Deletion of the yiaMNO transporter genes affects the growth characteristics of Escherichia coli K-12. Microbiology.

[33] Plantinga TH, Van Der Does C, Badia J, Aguilar J, Konings WN (2004). Functional characterization of the Escherichia coli K-12 yiaMNO transport protein genes. Mol Membr Biol.

[34] Novichkov PS, Kazakov AE, Ravcheev DA, Leyn SA, Kovaleva GY (2013). RegPrecise 3.0 – A resource for genome-scale exploration of transcriptional regulation in bacteria. BMC Genomics.

