# Supplementary material for: The Salmonella enterica serovar Typhimurium virulence factor STM3169 is a hexuronic acid binding protein component of a TRAP transporter
Source: Microbiology (Reading). 2020 Sep 7;166(10):981–7. doi: 10.1099/mic.0.000967 (PMC7660916; doi:10.1099/mic.0.000967)
Supplement: Supplementary material 1 [file mic-166-981-s001.pdf]

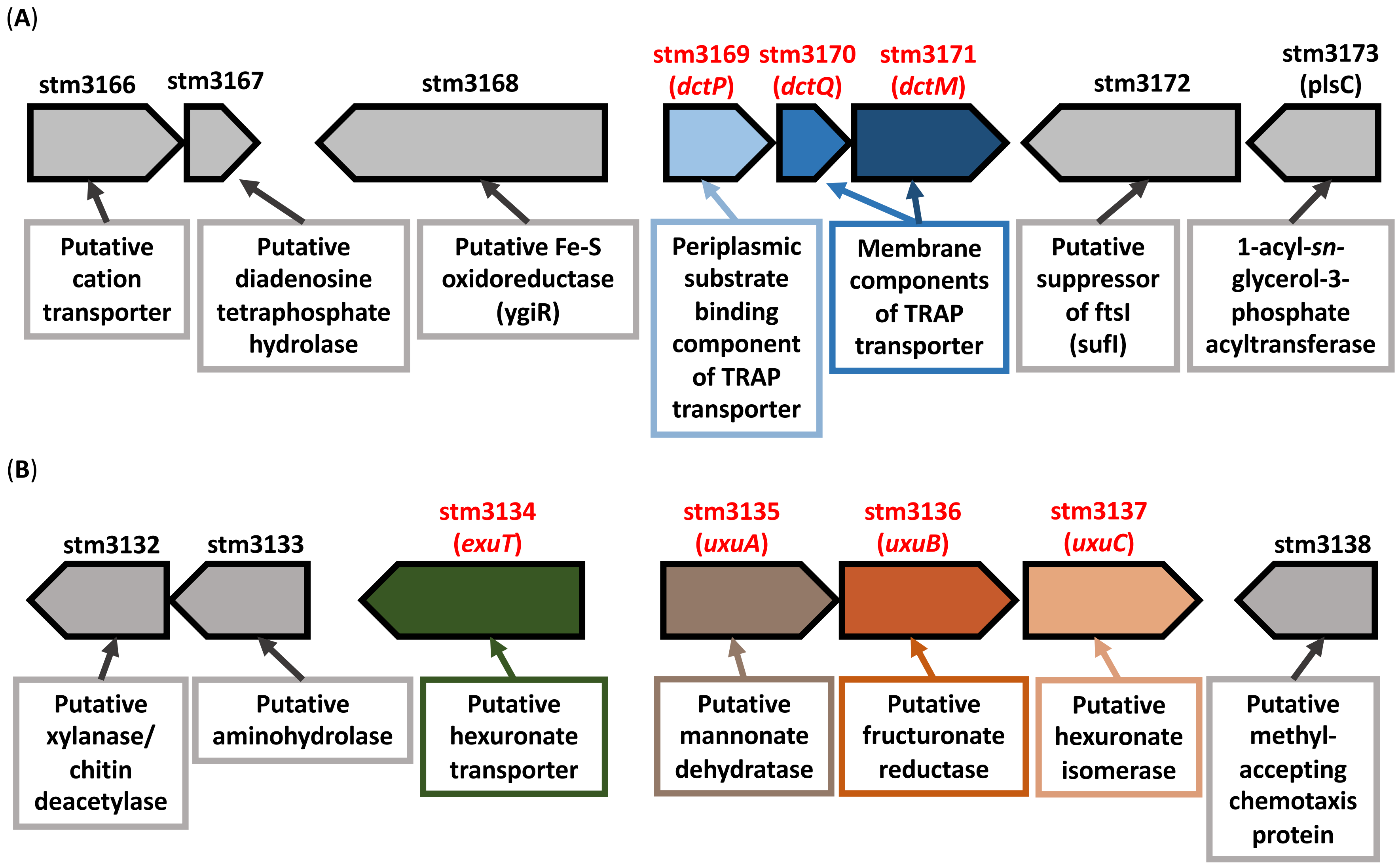

Figure S1. **Genome contexts** of the (**A**) *stm3169-stm3171* and (**B**) *stm3135-stm3137* operons. The transporter and catabolic gene positions and their neighbouring genes were identified using MicrobesOnline. Each gene is identified by their ordered locus name and experimentally verified or putative functions and names. Names of genes of interest are in red with neighbouring genes in black.

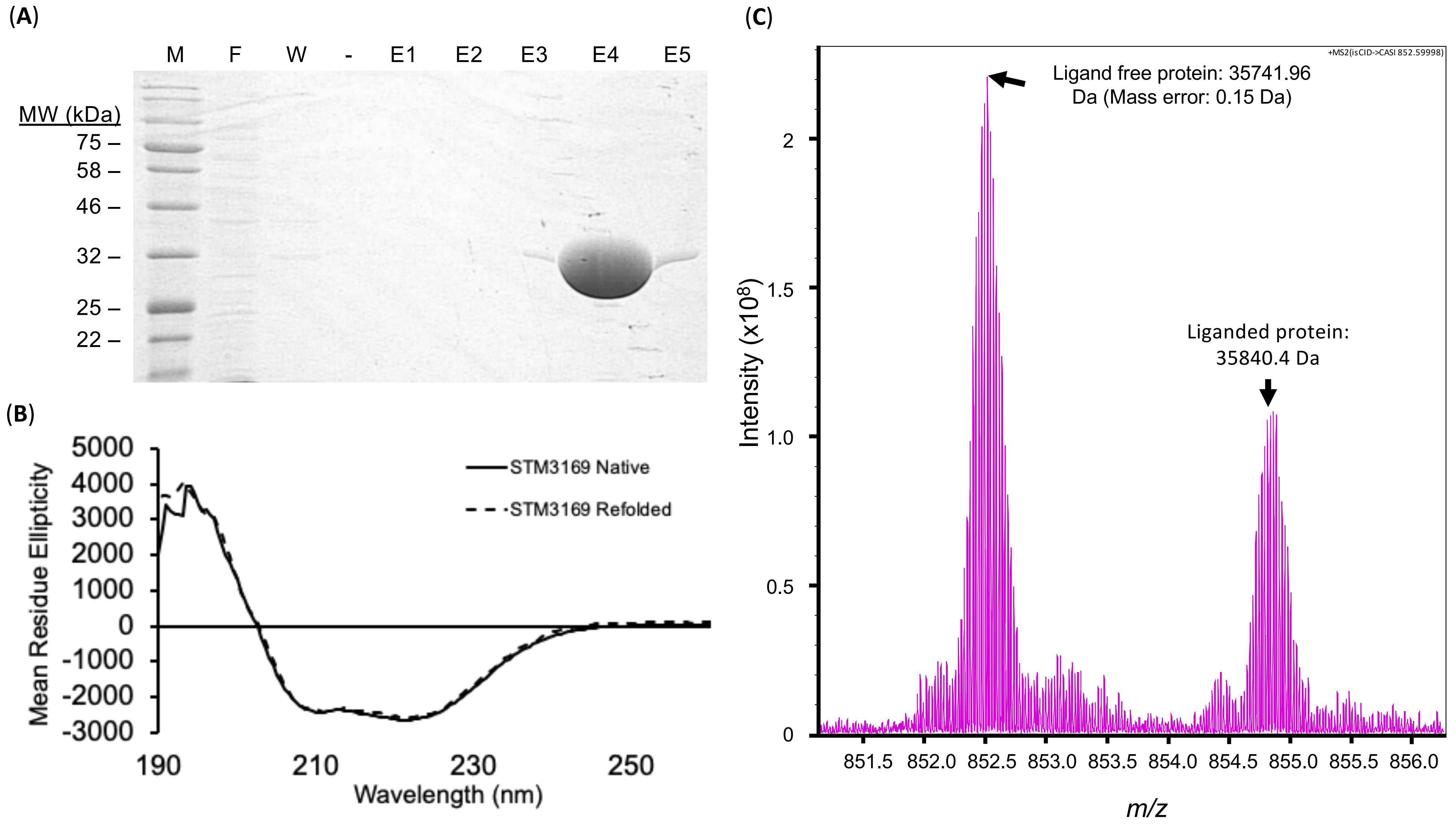

***Figure S2*. Assessing the quality of the purified STM3169.** (**A**) Coomassie stained gel of the Ni^{2+} affinity purification showing F, the flow through, W, the wash through and elution fractions E1-5. (**B**) Circular dichroism (CD) spectra of the natively folded protein and the protein refolded after denaturation using guanidine hydrochloride. (**C**) MS spectrum of STM3169 at the 42+ charge state. The spectra was analysed with and the molecular masses were deconvoluted using DataAnalysis v4.0 (Bruker Daltonics).
